# *Helicobacter pylori*-Induced Signaling Pathways Contribute to Intestinal Metaplasia and Gastric Carcinogenesis

**DOI:** 10.1155/2015/737621

**Published:** 2015-05-10

**Authors:** Soichiro Sue, Wataru Shibata, Shin Maeda

**Affiliations:** Department of Gastroenterology, Yokohama City University, 3-9 Fukuura, Kanazawa-ku, Yokohama 236-0004, Japan

## Abstract

*Helicobacter pylori* (*H. pylori*) induces chronic gastric inflammation, atrophic gastritis, intestinal metaplasia, and cancer. Although the risk of gastric cancer increases exponentially with the extent of atrophic gastritis, the precise mechanisms of gastric carcinogenesis have not been fully elucidated. *H. pylori* induces genetic and epigenetic changes in gastric epithelial cells through activating intracellular signaling pathways in a cagPAI-dependent manner. *H. pylori* eventually induces gastric cancer with chromosomal instability (CIN) or microsatellite instability (MSI), which are classified as two major subtypes of gastric cancer. Elucidation of the precise mechanisms of gastric carcinogenesis will also be important for cancer therapy.

## 1. Introduction (Figure 1)

Gastric cancer is the world's third leading cause of cancer-related death [1]. It is well known that the majority of gastric cancers are associated with* Helicobacter pylori* (*H. pylori*) infection [2]. Normal gastric mucosa, chronic superficial gastritis, atrophic gastritis, intestinal metaplasia, dysplasia, and adenocarcinoma are the chain of events with* H. pylori*. Atrophic gastritis and intestinal metaplasia exponentially increase the risk of developing gastric cancer (90-fold) [3]. Recent data from The Cancer Genome Atlas (TCGA) project led to the proposal of three subtypes of gastric cancer that were associated with* H. pylori*: (1) tumors with chromosomal instability (CIN), which display marked aneuploidy and focal amplification of receptor tyrosine kinases; (2) microsatellite unstable tumors (MSI), which have elevated rates of mutation, including mutations in genes encoding targetable oncogenic signaling proteins; and (3) genomically stable tumors (GS), which are enriched for the diffuse histological variant and fusions involving RHO-family GTPase-activating proteins or mutations of RHOA [4]. This review discusses pathogenesis and intracellular signaling pathways that are associated with* H. pylori* infection, which result in chronic inflammation, intestinal metaplasia, and gastric cancer.

## 2. *H. pylori*-Induced Chronic Inflammation and Intestinal Metaplasia (Figure 2)

### 2.1. CDX1/2: Key Factors for Gastric Carcinogenesis and Intestinal Metaplasia

Gastric intestinal metaplasia (IM) is considered to be a preneoplastic lesion of the stomach consisting of the transdifferentiation of the gastric mucosa into an intestinal phenotype, both morphologically and functionally [5]. Two types of IM: complete type and incomplete type are defined [6]. Complete IM express only intestinal mucin marker (MUC2), whereas incomplete IM express both intestinal and gastric mucin marker (MUC5AC) in single cell level [7]. The Wnt target genes CDX1 and CDX2 are thought to play a pivotal role in establishing and maintaining intestinal metaplasia and carcinogenesis, due to the observation that the intestinal phenotype is induced in cdx1 or cdx2 transgenic mice and that the intestinal-type adenocarcinoma is induced in cdx2 transgenic mice [5]. Several mechanisms of how CDXs contribute to the development of intestinal metaplasia have been reported. In a mouse model expressing intestine-specific homeobox genes, CDX1 transgenic mice developed a complete form of intestinal metaplasia, representing absorptional epithelial cells, goblet cells, gastrointestinal endocrine cells, and Paneth cells, while the characteristics of gastric mucosa completely disappeared [8]. In addition, Fujii et al. reported recently that CDX1 induces stemness-associated reprogramming factors, KLF5 and SALL4, suggesting that CDXs directly contribute to the development of gastric intestinal metaplasia. CDX1-induced KLF5 and SALL4 converted gastric epithelial cells into tissue stem-like progenitor cells, which then transdifferentiated into intestinal epithelial cells. A requirement for transition of intestinal metaplasia into dedifferentiated stem/progenitor-like cells, which share properties in common with cancer stem cells, may underlie the predisposition of intestinal metaplasia to neoplastic transformation [9]. Taken together, CDX1-induced dedifferentiated stem/progenitor-like cells in incomplete type IM may be essential for development of a preneoplastic lesion and may explain the diversity of gastric cancer.

In CDX2 transgenic mice, the expression of Shh, a morphogen associated with differentiation of the parietal cells of the stomach, was completely lost, both at the RNA level and when examined by immunohistochemistry [10]. Furthermore, the expression of Shh was decreased in the human intestinal metaplastic mucosa [11]. These phenomena support a key role for CDXs in the development of atrophic gastritis, intestinal metaplasia, and carcinogenesis.

In terms of signaling pathways, the Wnt and BMPs/SMAD4 pathways are both associated with the expression of CDX1/2. In addition to being a direct transcriptional target of the Wnt/*β*-catenin signaling pathway during mouse gut development, CDX1 is also induced by cag-positive* H. pylori* infection [9, 12]. BMPs/SMAD4 is known to be a fundamental pathway for the development of intestinal epithelium; it is upregulated upon* H. pylori* infection and thereafter induces the expression of the downstream target CDX2, as well as the downregulation of SOX2, an inhibitor of CDX2 [13–15]. CDX2 regulate MUC2 [16] by binding to enhancer sequences [17].

### 2.2. Genetic Alteration and Gene Expression in Intestinal Metaplasia

Gene alteration, such as aneuploidy of chromosomes [18], P53 mutations (38–45%) [19–22], P53 deletion (60%) [18], microsatellite instability (27%) [23], and mitochondrial microsatellite instability (33%) [24] were detected in IM. P53 mutations were mostly in incomplete type [20, 21]. Microsatellite instabilities were all in incomplete type [25]. Gene expression, such as MUC2 [6], LI-cadherin [26], KLF4 [27], intestinal trefoil factor (TFF3) [28], sucrose-isomaltase [29], villin [7], CD10 [30], and defensing [31], increased in IM. MUC2 is regulated by CDX2 [16, 32]. On the other hand, gene expression, such as Sonic hedgehog (Shh) [33], SOX2 [14], RUNX3 [34], and TFF1 and TFF2 [28], decreased in IM. Shh is particularly decreased in incomplete IM type [11].

Alteration of these gastric and intestinal phenotype markers was observed at the cellular level, as well as at the glandular level. In fact, neuroendocrine cells also showed intestinalization along with their exocrine counterparts. In animal models, incomplete type intestinal metaplasia appears first and then progresses to the complete type. In summary, intestinal metaplasia may be caused by the gradual intestinalization of stem/progenitor cells from the incomplete to the complete type [35].

## 3. *H. pylori*-Induced Genetic Changes

Several reports have suggested that* H. pylori* infection caused genetic alterations in gastric epithelial cells, mostly through the induction of reactive oxygen species (ROS) [36]. Matsumoto et al. reported that* H. pylori* induced aberrant expression of activation-induced cytidine deaminase (AID), known as an editor of DNA and RNA. AID was reported to cause mutations in the P53 and APC genes in gastric epithelial cells, relevant to the development of adenocarcinoma [37]. AID hypermutates immunoglobulin genes in B cell genome, contributing to variety acquisition of immunoglobulin. AID also target oncogenes, leading to B cell malignancy [38]. In addition, various cancers develop in AID transgenic mice, including gastric cancer [39]. In Matsumoto's report,* H. pylori* strongly induced AID expression in human gastric epithelial cells, through activation of the NF-*κ*B pathway, and induced mutation of p53. As mutation of p53 was inhibited by blocking AID, p53 mutation induced by* H. pylori* mostly depends on AID. Since AID was upregulated via activation of the NF-*κ*B pathway, proinflammatory cytokines—such as TNF-*α* or IL-1*β*—in gastric inflammation also reinforce the onset of AID as well as the direct stimulation of* H. pylori* in gastric epithelial cells [40].

## 4. *H. pylori*-Induced Epigenetic Changes

### 4.1. DNA Methylation Induced by* H. pylori* Infection

It has been reported that* H. pylori* could cause DNA methylation of many genes in gastric epithelial cells. Mongolian gerbils were infected with* H. pylori* and DNA methylation levels in the gastric mucosa were analyzed over time. Methylation levels were increased in the persistent infection group depending on the duration of infection [41–43]. Accordingly,* H. pylori* eradication led to a dramatic decrease in methylation levels [44, 45]. Since DNA methylation remained after infection with* H. pylori* and methylation could be inhibited with an immunosuppressive drug, it can be concluded that the inflammatory reaction induced by* H. pylori* infection, and not the presence of the bacterium itself, is more important in the process of DNA methylation [43].* H. pylori* infection causes gastric mucosal inflammation responses, resulting in upregulation of IL-1*β* or Nos2, which in turn induce aberrant DNA methylation [46]. Several studies found that aberrant DNA methylation in gastric biopsies from* H. pylori*-positive patients correlated with a greater risk of developing gastric cancer [43, 47], suggesting that* H. pylori*-associated inflammation and subsequent induction of DNA methylation could have a potential role in gastric carcinogenesis. A large number of genes with different biological functions have been found to be methylated in gastric carcinogenesis. Among these, methylation of a DNA repair gene, MLH1, may play an important role in gastric carcinogenesis in MSI-positive gastric cancer, since MLH1 is silenced in this type of cancer.

### 4.2. *H. pylori* and Gastric CIMP

Aberrant DNA methylation in cancer encompasses global hypomethylation and regional hypermethylation, which are thought to be associated with genomic instability and inactivation of tumor-suppressor genes [48]. However, regional hypermethylation refers to the aberrant methylation of normally unmethylated sequences, most of which are clusters of CpG sites, denoted as CpG islands. The strong relationship between CIMP and MSI suggests that CIMP may be related to gene mutation. In fact,* H. pylori* infection significantly elevated the rate of CIMP positivity [49], suggesting that* H. pylori* caused aberrant DNA hypermethylation of specific genes, followed by induction of CIMP during gastric carcinogenesis.

## 5. Changes in Signaling Pathways Induced by* H. pylori* Infection

Numerous signaling pathways mediated by* H. pylori* are reportedly dependent on the cag pathogenicity island (cagPAI), especially the cagA gene. Elucidation of the signaling pathways activated by* H. pylori* infection may be important for the identification of targets for treatment.

### 5.1. NF-*κ*B Pathway (Figure 3)

NF-*κ*B is one of the major transcription factors that regulates inflammation and is constitutively activated in some gastric cancers [50].* H. pylori* activates NF-*κ*B in the gastric mucosa via cagPAI-dependent and cagPAI-independent pathways.* H. pylori* cag-positive strains deliver the certain protein into host cells via the cag PAI-encoded type IV secretion system (T4SS) [51–53]. The certain protein is thought to be injected into host epithelial cells where it interacts with TRAF6 and TAK1 to activate IKK. The IKK-complex contains two highly homologous kinase subunits, IKK*α* and IKK*β*, in addition to the regulatory subunit NF-*κ*B essential modulator (NEMO). The key factor required for activation of this pathway is still unknown. One possible explanation is that peptidoglycan injected into cells via the T4SS stimulates Nod1 activity leading to NF-*κ*B activation [54]. During cag-independent activation of intracellular signaling, host immune cells are stimulated by lipopolysaccharide (LPS) produced by* H. pylori* via TLR pathways, followed by activation of the NF-*κ*B pathway [55–58]. Among certain proteins injected into host cells, CagA is probably indispensable in the induction of an inflammatory reaction, as it has been reported that a CagA-knockout of* H. pylori* was unable to induce severe inflammation in Mongolian gerbils model [59, 60]. It has also been reported that overexpression of CagA induced NF-*κ*B activation with subsequent IL-8 production [61].

NF-*κ*B activation induces the release of proinflammatory cytokines, such as tumor necrosis factor- (TNF-) *α*, interleukin- (IL-) 1*β*, and IL-6 [62–65]. NF-*κ*B also regulates other molecules that are involved in the chemokine response (IL-8, MCP-1), blockade of apoptosis (cIAPs, c-FLIP, A20, and BclX), angiogenesis (VEGF, IL-8), and invasion (MMP-2, MMP-9). All these factors may be related to carcinogenesis [66], and we have focused on inhibition of NF-*κ*B as a potential avenue to inhibit cancer, by controlling the degree of gastritis caused by* H. pylori* infection [67].

### 5.2. The IL-6- (IL-11-) STAT3-CDX2 Pathway (Figure 4)

It has been reported that the proinflammatory cytokine IL-6, which is upregulated upon* H. pylori* infection in the gastric mucosa, contributes to gastric tumorigenesis [68]. IL-6 binds to the *α*-subunit of its specific receptor, associates with gp130 homodimers at the cell membrane, and activates two main signaling pathways: SHP-2/ERK and JAK/STAT. In mouse models, gp130F/F mice spontaneously develop gastric inflammation and intestinal-type gastric tumors [69]. gp130 is IL-6 family receptor signaling subunit, and IL-6 family gp130 driver IL-11 drives hyperactivation of STAT3 contributing gastric phenotype. Gp130 F/F carries a knock-in mutation in gp130 [70]. The final step in the SHP-2/ERK pathway is gene regulation by the transcription factor AP-1, whereas in the JAK/STAT pathway, phosphorylated STAT3 dimers translocate to the nucleus and activate the transcription of target genes [71].

The* H. pylori* protein CagA recruits SHP2 to gp130; phosphorylated CagA shows enhanced SHP2 binding activity and ERK1/2 phosphorylation, whereas unphosphorylated CagA preferentially activates STAT3 [72]. SHP2/ERK signaling may lead to mucosal inflammation and carcinogenesis [73]. Phosphorylated STAT3 induces expression of genes that promote angiogenesis (e.g., VEGF), cell-cycle progression (e.g., cyclinD1), and cell survival (e.g., Bcl/xL, survivin) [69]. Mice in which gp130 is mutated and the STAT3 pathway is activated develop gastric cancer [74]. Taken together, these findings suggest that the IL-6/STAT3 pathway plays key roles in gastric carcinogenesis, not only via the IL6- (IL11-) STAT3-CDX2 pathway resulting in induction of intestinal metaplasia, but also via the unphosphorylated CagA/SHP2/ERK1/2 pathway, leading to induction of gastric epithelial proliferation and carcinogenesis.

### 5.3. c-Myc/p21/ERK-MAPK Pathway

It was reported that cagA coupled with PAR1 (MARK) phosphorylation resulted in disruption of tight junctions and the cellular polarity of epithelial cells [75]. CagA, which invaded epithelial cells, bound to PAR1 and stimulated the GEF (guanine nucleotide exchange factor)–H1–RhoA–ROCK (RhoA-associated kinase)–c-Myc–microRNA–p21 axis; this was caused by the liberation of cigulin from the GEF-H1-cingulin complex, followed by induction of miR-17 and miR-20a by c-Myc activation, resulting in inhibition of accumulation of p21. In other words, p21 causes cellular senescence by inhibiting activation of ERK-MAPK, and CagA causes abnormal cell proliferation by inhibiting p21, thus contributing to gastric carcinogenesis [76].

### 5.4. TLR Signaling

Toll-like receptors (TLRs) are a key family of microbial sensors of the host innate and adaptive immune systems [77]. For instance, genetic ablation of signaling adaptor MyD88 and the common TLR in mice alleviates intestinal tumorigenesis induced in ApcMin/+mice [78]. TLRs expressed in epithelial can promote noninflammatory epithelial responses including migration, cell survival, proliferation [79], and angiogenesis [80]. In the setting of* H. pylori* infection, gene expression of TLR2 and TLR4 is elevated in* H. pylori*-positive gastric patients [81], and TLR2 and TLR4 gene polymorphisms are associated with an increased risk of gastric cancer [82]. STAT3 directly upregulates epithelial expression of TLR2 in gastric tumors. Genetic and therapeutic targeting of TLR2 inhibited gastric tumorigenesis (but not inflammation), characterized by reduced proliferation and increased apoptosis of gastric epithelial cells [83]. Increased STAT3 pathway activation and TLR2 expression were also associated with poor survival in gastric cancer patients.

### 5.5. ROS/ASK1/JNK Pathway

ASK1 is reported to be one of the key players in the regulation of* H. pylori*-related cellular responses in gastric epithelial cells. ASK1 is involved in cellular responses induced by* H. pylori*, such as apoptosis and cytokine production. Furthermore, ASK1 and TAK1 have reciprocal interactions and differentially regulate the activation of downstream molecules, such as JNK, p38, and NF-*κ*B [84].

JNK, which can be activated by* H. pylori* infection via both ASK1 and TAK1, plays an important role in gastric carcinogenesis. In human gastric cancer, the extent of activated JNK observed by immunostaining is approximately 30–40%, whereas activation was observed in almost all cases of* H. pylori*-infected gastric mucosa. In a mouse MNU gastric carcinogenic model, number and diameter of the tumor cells were significantly decreased in JNK knockout mice compared to WT mice [85]. The effect on cellular proliferation was also examined in vitro; it was found that cellular proliferation was inhibited by using a JNK inhibitor or an siRNA to knock down JNK expression. Constitutive activation of JNK is proposed to be due to a positive feedback loop: ASK/JNK/CyclinD1/Rb phosphorylation/ASK protein upregulation [86].

## 6. *H. pylori*-Induced Chronic Inflammation and Innate/Adaptive Immunity

As mentioned above, after infecting* H. pylori *on gastric epithelial cells, it can affect not only the proliferation of gastric epithelial cells, but also the activation of intracellular signaling, and that leads to perturbing the host's innate and adaptive immune system [87]. Among the inflammatory reactions induced by* H. pylori* infection, innate immune system, represented by infiltration of neutrophils and macrophages, plays key roles in production of proinflammatory cytokines/chemokines, which promote chronic inflammation [67]. On the other hand, adaptive immune system plays roles not only to produce proinflammatory cytokines and cytotoxic reaction to bacterium directory, but also in induction of anti-inflammatory cytokines, such as IL-10, to suppress the cytotoxic function of effector T cells, which enables the bacteria to evade immune system, resulting in chronic infection [88].

## 7. Conclusions and Future Perspectives

Since chronic inflammation can cause epithelial cell disturbance, the early eradication of* H. pylori *could provide a basic solution for prevention of gastric carcinogenesis caused by* H. pylori*. Accordingly, all patients in Japan with* H. pylori*-related gastritis are being recommended for eradication methods to decrease the risk of gastric cancer. However, as a considerable proportion of patients remain to be irreversible “field cancerization,” where the cancer-causing case does not cut off after* H. pylori* sanitization, precancerous intestinal metaplasia even after* H. pylori* eradication, it is difficult to identify those at high risk of gastric cancer. Regarding the treatment of advanced gastric cancer, HER2 has emerged as a successful molecular target, and the treatment of other RTK/RAS amplifications complies with the concept of oncogene addiction, dependency of cancer on one or a few genes for maintenance of the malignant phenotype. Elucidation of the mechanism of gastric carcinogenesis associated with* H. pylori* will aid the development of further targeted therapies, which will be accompanied by the advent of personalized cancer medicine, a field that is developing rapidly.

## Figures and Tables

**Figure 1:**
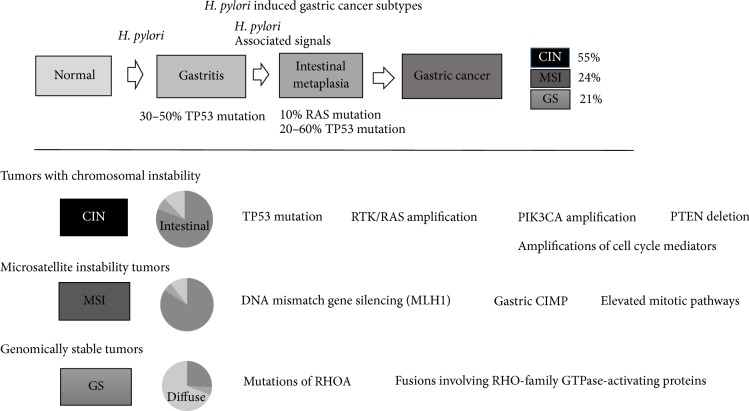
Summary of the three subtypes of gastric cancer that were associated with* H. pylori*.

**Figure 2:**
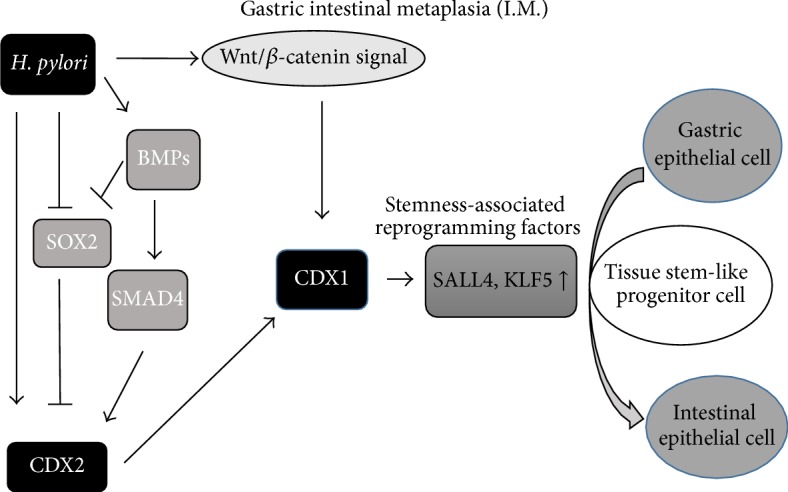
Putative mechanism for inducing intestinal metaplasia by* H. pylori.*

**Figure 3:**
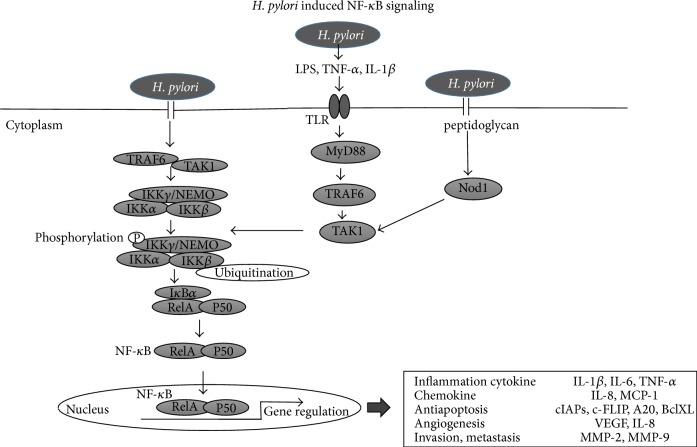
*H. pylori* induced NF-*κ*B signaling.

**Figure 4:**
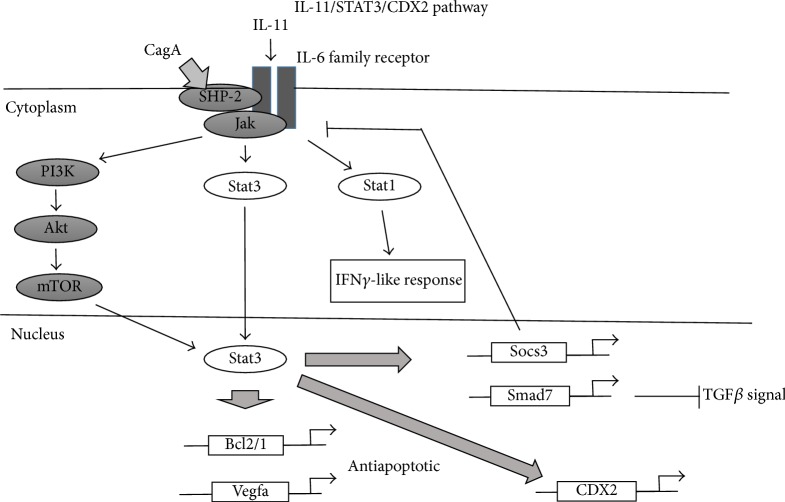
Scheme of the IL-11/STAT3/CDX2 pathway.
